# Exploring the Need for Psychiatry Sub-Specialization in Nepal: A Cross-sectional Survey Among Psychiatrists

**DOI:** 10.31729/jnma.9153

**Published:** 2025-07-31

**Authors:** Omkar Dhungel, Reet Poudel, Yujal Man Singh, Bharat Khadka, Ayushma Shah, Suman Prasad Adhikari

**Affiliations:** 1 Shree Birendra Hospital, Chhauni, Kathmandu, Nepal

**Keywords:** *Doctor of Medicine (DM)*, *fellowship*, *psychiatry*, *sub-specialization*

## Abstract

**Introduction:**

The complexities in the identification and management of mental disorders have demanded sub-specialization. In Nepal, the number of psychiatrists still does not meet the minimum requirement of the psychiatrist-population ratio. There is a dilemma regarding the need for further subspecialization in psychiatry. This study aimed to assess the perceived need for psychiatry sub-specialization courses among Nepalese psychiatrists.

**Methods:**

This cross-sectional descriptive study was conducted via an online survey (using Google Forms) among Nepalese psychiatrists and psychiatry residents. Convenience and snowball sampling was used. Data was analysed using Statistical Package for Social Sciences (SPSS), presented in frequency and percentage.

**Results:**

Among 165 respondents, 99 (60%) were male, 105 (63.60%) were between 30-40 years of age, 125 (75.80%) were psychiatrists, and 138 (83.60%) expressed a need for further sub-specialization, although only 102 (60%) were interested. The primary reason for not pursuing sub-specialization was the need for general psychiatrists. Fellowship in addiction psychiatry was the most frequently selected sub-specialty.

**Conclusions:**

Most of the participants stated that there is a need for pyschiatry sub-specialization in Nepal.

## INTRODUCTION

The formal psychiatry outpatient treatment started in Nepal in 1961 A.D.^1^ and the first Doctor of Medicine (MD) course was started in 1997.^2^ The complexities in the identification and management of mental disorders have demanded a subspecialty.^3^ Currently, there are 240 registered psychiatrists in the Psychiatrists’ Association of Nepal (PAN).^4^

The number of psychiatrists still does not meet the minimum requirement of one for 10,000 people.^5^ On the other hand, the current context is demanding sub-specialization in all the medical fields and psychiatry is not untouched. At the same time, there is a dilemma among early-career psychiatrists in Nepal about the necessity to pursue further sub-specialty.

The objective of this study was to find out the perceived need for psychiatry sub-specialization courses among Nepalese psychiatrists and psychiatry residents, and the factors affecting the perceived need. As per our search in Google Scholar, PubMed, and NepJOL, there has been no such published study in Nepal.

## METHODS

The study was a cross-sectional descriptive study, conducted via an online survey among Nepali psychiatrists based in Nepal or abroad and Nepali psychiatry residents studying in Nepal. The study protocol was according to the ethical standards and was approved from the Institution Review Committee (Reg. No: 1170). This study employed convenience and snowball sampling methods. Psychiatry residents in Nepal and Nepali psychiatrists above 18 years were included in the study. The exclusion criteria were participants not giving written consent for participation or participants unable to understand or respond to the survey due to language, cognitive impairment, or other reasons.

The required sample size was calculated as 165 using the finite population correction formula, assuming a prevalence of 50%, a 95% confidence level, and a 5% margin of error. The total population was 280, comprising 240 registered psychiatrists and 40 psychiatry residents.

A self-designed pro forma was prepared by a team of psychiatrists with respect to the needs of the study. It includes relevant and appropriately framed questions made in such a way as to fulfill the requirements of the social demographic profile and the perceived need for sub-specialization among Nepali psychiatrists. The variables included were age, gender, country (Nepal or abroad) from which MD psychiatry was completed, duration after completion of MD psychiatry, any other qualifications after MD psychiatry, current working position, need for sub-specialization, type of specialization, preferred duration of specialization, most needed specializations, and the preferred country (Nepal or abroad) for specialization.

The data was collected via an online platform on Google Forms. Individual psychiatrists and psychiatry residents were called over the phone and briefed about the survey by an author to minimize potential response bias and ensure that participants fully understand the study’s purpose, scope, and confidentiality before providing consent. Then they were provided a link to the Google form in their email or WhatsApp on day zero, two weeks, and six weeks. One email address could fill the form only once, and they were informed to fill the form one time only. There was written informed consent for participation in the study at the beginning of the Google form and an answer to the question, “Do you want to continue? (Yes/No)”, where “Yes” implied the acceptance of the informed written consent and “No” implied the rejection. They were asked to send an email, WhatsApp message, or call over the phone if they had any queries.

Participants completed the questionnaires privately without their names in the submission, so that they could provide honest responses without skepticism of being judged. After the participants completed filling out the Google form (by two months, 10th January to 9th March 2025) and submitted it, the data was available to the author for analysis. Data was analyzed using SPSS.

## RESULTS

The total number of participants to complete the survey was 165. Of these, 99 (60%) were male and 66 (40%) were female. Participants aged 30 to 40 years were 105 (63.60%). Of the total participants, psychiatrists were 125 (75.80%) and psychiatry residents were 40 (24.20%). Among the psychiatrists, 93 (74.40%) completed their MD from Nepal and 32 (25.60% from abroad. In our study, among 125 psychiatrists, 116 (92.80%) were practicing psychiatry in Nepal, and others abroad. Psychiatrists without additional sub-specialty training after MD Psychiatry were working across various settings, including teaching hospitals as faculty.(Table 1).

Out of total, 138 (83.60%) expressed that there is a need for further sub-specialization study in psychiatry, 102 (61.80%) were interested in doing the sub-specialization. Out of 63 (38.20%) respondents who responded the reasons for not doing the sub-specialization, 19 (30.15%) stated that Nepal needs more general psychiatrists (Table 2).

**Table 1:** Duration after completion of MD, further subspecialty, and current position of psychiatrists (n = 125).

Variable	n (Percent)
Duration after completion of MD	
< 1 year	14 (11.20)
1 – 5 years	44 (35.20)
6 – 10 years	32 (25.60)
> 10 years	35 (28.00)
Further sub-specialty after MD	
None	96 (76.80)
Fellowship	12 (9.60)
DM	2 (1.60)
PhD	5 (4.00)
Others	10 (8.00)
Current position	
Faculty in a teaching hospital	61 (48.80)
Works in a hospital	49 (39.20)
Works in a private clinic	14 (11.20)
Not practicing	1 (0.80)

**Table 2:** Reasons for not doing the sub-specialization (n = 63)

Reasons	n (%)
Psychiatry in Nepal needs more general psychiatrists	19 (30.15)
Psychiatry in Nepal is not ready for sub-specialization	14 (22.22)
It is too late for further sub-specialization	13 (20.63)
Due to family reasons	3 (4.76)
I have done	14 (22.22)

**Table 3:** Relationship between different variables with whether they would pursue further sub-specialization (n=165).

	Further Specialization	
Variable	Yes n(%)	No
Gender		
Male	55(33.33%)	44(26.67%
Female	47(28.48%)	19(11.52%
Academic status		
Psychiatrist	72(43.64%)	53(32.12%)
Resident	30(18.18%	10(6.06%)
Duration after completion of MD		
<10 years	58 (35.15%)	14 (8.48%)
>10 years	32 (19.39%)	21 (12.73%)
Current position/working in		
Private clinics	4 (2.42%)	10 (6.06%)
Hospital	67 (40.61%)	4 (2.42%)

**Figure 1:**
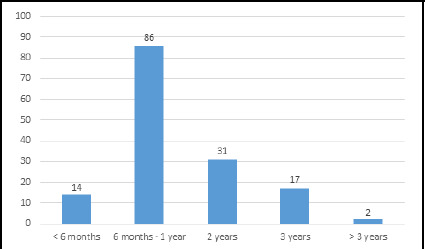
The most preferred duration of sub-specialization (n = 150).

**Figure 2:**
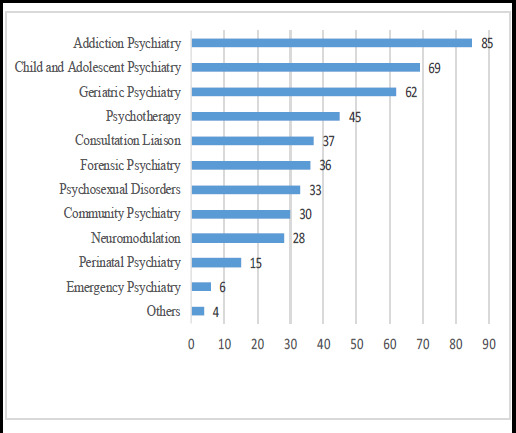
The most needed sub-specialization after MD psychiatry (n = 150 respondents selecting three categories).

Among 150 respondents, fellowship was the preferred subspecialization type 102 (68%) (Figure 1), with a duration of six months to one year (Figure 2). Addiction Psychiatry was preferred by 85 (56.67%), followed by Child and Adolescent Psychiatry by 69 (46%) (Figure 2). Among 102 participants interested in further study, 52 (50.98%) preferred to study in Nepal, while 50 (49.02%) preferred abroad.

Among the total respondents, 55 (33.33%) males and 47 (28.48%) females reported willingness to pursue further sub-specialization. In terms of academic status, 72 (43.64%) psychiatrists and 30 (18.18%) residents expressed interest. A total of 58 (35.15%) participants with less than 10 years since completion of MD indicated willingness to pursue further sub-specialization. Similarly, 67 (40.61%) of those working in hospitals reported willingness to pursue further sub-specialization.

## DISCUSSION

This survey was conducted to assess the perspectives of psychiatrists and psychiatry residents regarding the need for sub-specialization in psychiatry, preferred courses, and course duration. Among 165 participants, 40 were residents and 125 were psychiatrists. Among the psychiatrists, 29 (23.2%) had done some specialization after MD, and only 2 (1.6%) had completed a DM degree. A similar survey done in India showed that 24 (16.9%) had a DM degree.^3^ This difference could be due to the availability of DM courses in psychiatry in India for decades, while such programs are yet to be introduced in Nepal.^3^

In this study, 63 (32.2%) participants stated they did not plan to pursue further sub-specialization. Of them, more than half (30.15%) believed that psychiatry in Nepal needs more general psychiatrists rather than sub-specialized manpower. As mentioned, the number of general psychiatrists per population is still below the requirement.^5^ They are also concentrated within and around the capital city, resulting in a considerable gap in mental health services across Nepal.

Although psychiatrists generally perceived a high need for addiction psychiatry, followed by child and adolescent psychiatry, in this study, both DM degree holders are child and adolescent psychiatrists based in Kathmandu. It may be due to our society reflecting prejudices and considering substance-use to be a self-indulgent problem of the emotionally weak-willed,^6^ and to enhance positive emotions and social experiences.^7^ This may also be due to personal preference or an ever-expanding emotional and behavioral problem in children.^8^ Similar to the study in India,^3^ the most preferred sub-specialization program was a fellowship of duration six months to one year. This preference could be due to the time factor and the financial factor. Lengthy courses like DM or PhD demand a long duration with limited monthly earnings. Even in the USA, 12-month fellowship courses in psychiatry are accredited by the Accreditation Council for Graduate Medical Education (ACGME).^3^ In addition, psychiatrists might be reluctant to limit their skills to a specific subspecialty after extensive training in DM courses.^3^

It was found that the residents opted to pursue further subspecialization more than psychiatrists. This may be due to the growing interest among young doctors in sub-specialization, as in other fields of medicine. Residents might be more enthusiastic about further study than consultants. Consultant psychiatrists and those who have completed MD for more than ten years have less interest in further specialization, which might be due to involvement in their job and career, stable earnings, and family commitments. They might not be able to spare one year or more for further study. Psychiatrists working in hospitals were more interested in sub-specialization than those working in private clinics. This might be due to continuous encounters with diverse, complicated, and treatment-resistant cases, discussion in ward settings, and their close follow-up of progress, and a more academic setup. With these findings and discussion, the authors tried to evaluate the situation of sub-specialization in psychiatry in Nepal.

With time, managing mental illness has moved from trephination, purging, bloodletting, and isolation to asylum and finally to hospitals and community care.^9^ Psychiatry was separated from the mainstream of general medicine in the early 19th century and has established itself as a major discipline.^10^ It has evolved with new advancements in understanding and practice.

In Low and Middle Income Countries (LMIC), approximately four out of five people with some mental illness do not seek professional help and receive no medical treatment.^11^ The scenario is similar or more severe in Nepal due to lack of human and material resources, and people’s perception.^12,13^ Though the community mental health program was started four decades ago, it has been only three decades since the psychotic patients were moved from jails to hospitals.^2^ So, the modern mental health practice is still young in Nepal. The mental health services in Nepal have been streamlined since the 1960s.^2^ The first Doctor of Medicine (MD) course in Nepal was started at the Institute of Medicine, Tribhuvan University in 1997.^2^ There are around 500 beds for people with mental disorders (i.e., 1.5 beds per 100.0 people), 200 psychiatrists (0.68 psychiatrists per 100.0 people).^1^ This signifies, we are struggling in manpower and resources.

On the other hand, the complexities in the identification and management of mental disorders have demanded sub-specialty training programs. These sub-specialty programs in psychiatry have been in place in different countries, including India, for decades.^3^ After completion of a master’s degree in psychiatry for three or more years, a fellowship or Doctor of Medicine (DM) in those sub-specialties continues for another one to three years. Different sub-specialties in psychiatry include child and adolescent psychiatry, addiction psychiatry, forensic psychiatry, geriatric psychiatry, consultation-liaison psychiatry, psychotherapies, sexual medicine, sleep medicine, and others.^3^

The current context is demanding sub-specialization in all medical fields, and psychiatry is not untouched. At the same time, there is a dilemma among early-career psychiatrists in Nepal about the necessity to pursue further sub-specialty. In Nepal, where basic priorities in mental health are yet to be met, the rationale behind the need for sub-specialization is questionable. We need planning and prioritization in mental health, and every plan should be based on the country’s needs. Sub-specialization in psychiatry is undoubtedly a genuine idea for any country, but it should be prioritized only after basic mental health needs are fulfilled. The basic mental health needs in developing countries are to increase mental health awareness, to provide basic mental healthcare services, to decrease the economic burden, and to increase the interest among medical students in psychiatry.^10^ In low and middle-income countries, there are insufficient resources for sub-specialists, and a primary care approach is necessary. At the same time, a primary care approach does not supersede the need for specific area experts and tertiary consultations by those experts.

Sub-specialization may be sub-specialty dependent. In the surgical field, sub-specialization is associated with better outcomes, as mastery of different techniques is essential. However, in other branches of medicine, narrow expertise can be suboptimal. In the sub-specialty environment, there can be longer waiting times, over-testing, and overtreatment.^14^

Similar to other areas of medicine, psychiatry is growing rapidly, and it is almost impossible for one person to master all areas. The treatment would be more problem-focused if seen by someone who has expertise in specific psychiatric disorders, benefiting patient outcomes. At the same time, there is a possibility that the services become overly compartmentalized.^15^ This would create a dilemma for patients, who to see and where to go. As dual diagnosis is common in mental disorders,^15^ patients will have to visit multiple psychiatrists for holistic care. Thus, the stakeholders need to plan and act carefully, balancing the strategy to provide basic mental health services along with expert tertiary care in the days to come.

The study is subject to several limitations that need to be discussed. The sampling method used was convenience sampling, which could have led to selection bias. In-person data collection could have been better than an online survey, but any issues if identified were resolved through telephone calls or online messaging then and there. Though the participants were informed that the pro forma was short and could be finished within three minutes, there could be a response bias if participants quickly moved through the questions for the sake of completing the task. Selection bias could have occurred due to the under-representation of certain groups, such as senior psychiatrists, which was minimized by repeated reminder via phone call or message.

## CONCLUSIONS

Most of the respondent expressed that there is a need for further further sub-specialization in psychiatry. More than half were interested in doing sub-specialization. Most of the respondents who responded the reasons for not doing the sub-specialization stated that Nepal needs more general psychiatrists.

## Data Availability

The data are available from the corresponding author upon reasonable request
